# Fetal Alcohol Exposure and IQ at Age 8: Evidence from a Population-Based Birth-Cohort Study

**DOI:** 10.1371/journal.pone.0049407

**Published:** 2012-11-14

**Authors:** Sarah J. Lewis, Luisa Zuccolo, George Davey Smith, John Macleod, Santiago Rodriguez, Elizabeth S. Draper, Margaret Barrow, Rosa Alati, Kapil Sayal, Susan Ring, Jean Golding, Ron Gray

**Affiliations:** 1 School of Social and Community Medicine, University of Bristol, Bristol, United Kingdom; 2 MRC Centre for Causal Analyses in Translational Epidemiology, School of Social and Community Medicine, University of Bristol, Bristol, United Kingdom; 3 National Perinatal Epidemiology Unit, University of Oxford, Oxford, United Kingdom; 4 Department of Health Sciences, University of Leicester, Leicester, United Kingdom; 5 Clinical Genetics, University Hospitals of Leicester, Leicester, United Kingdom; 6 School of Population Health & Centre for Youth Substance Abuse Research, University of Queensland, Queensland, Australia; 7 Developmental Psychiatry, University of Nottingham, Nottingham, United Kingdom; Vanderbilt University, United States of America

## Abstract

**Background:**

Observational studies have generated conflicting evidence on the effects of moderate maternal alcohol consumption during pregnancy on offspring cognition mainly reflecting problems of confounding. Among mothers who drink during pregnancy fetal alcohol exposure is influenced not only by mother’s intake but also by genetic variants carried by both the mother and the fetus. Associations between children’s cognitive function and both maternal and child genotype at these loci can shed light on the effects of maternal alcohol consumption on offspring cognitive development.

**Methods:**

We used a large population based study of women recruited during pregnancy to determine whether genetic variants in alcohol metabolising genes in this cohort of women and their children were related to the child’s cognitive score (measured by the Weschler Intelligence Scale) at age 8.

**Findings:**

We found that four genetic variants in alcohol metabolising genes in 4167 children were strongly related to lower IQ at age 8, as was a risk allele score based on these 4 variants. This effect was only seen amongst the offspring of mothers who were moderate drinkers (1–6 units alcohol per week during pregnancy (per allele effect estimates were −1.80 (95% CI = −2.63 to −0.97) p = 0.00002, with no effect among children whose mothers abstained during pregnancy (0.16 (95%CI = −1.05 to 1.36) p = 0.80), p-value for interaction  = 0.009). A further genetic variant associated with alcohol metabolism in mothers was associated with their child’s IQ, but again only among mothers who drank during pregnancy.

## Introduction

The public health burden associated with alcohol use includes any adverse outcomes experienced by children whose mothers used alcohol during pregnancy. The deleterious effects of heavy maternal alcohol use on offspring outcomes are well established [1] however effects of more moderate use are less clear. Official guidelines on safe drinking during pregnancy appear contradictory on this point, with some advocating complete abstinence and others suggesting that moderate use is safe (http://www.icap.org/Table/InternationalGuidelinesOnDrinkingAndPregnancy).

A recent systematic review of findings from observational studies found no consistent evidence of adverse effects from low-to-moderate prenatal alcohol consumption [2], as did an even more recent study of a population based cohort examining this issue [3], [4].Interpreting observational evidence on effects of maternal alcohol use on offspring outcomes is complicated by the issue of confounding. In particular, complete abstinence from alcohol is often associated with other maternal characteristics that may adversely influence offspring outcomes [5] whilst moderate alcohol use is often associated with characteristics that may exert independent beneficial effects. Statistical adjustment for confounding of this nature is notoriously difficult. The alternative of a randomized controlled trial (RCT) would be unethical, unless this were an RCT of an intervention to stop drinking during pregnancy and then there would be uncertainty surrounding its effectiveness. Quasi experimental designs may be useful to progress the evidence in this area. One novel approach, Mendelian randomization, provides an alternative method for investigating the causal nature of early life influences on later diseases [6], [7]. Associations between genetic variants and disease are not generally susceptible to confounding by lifestyle factors [8] and genetic variants which influence exposure to alcohol by affecting the ability to metabolise alcohol, should not be subject to confounding by smoking, diet and other lifestyle factors.

The conversion of ethanol to acetaldehyde is catalysed primarily by a group of 5 alcohol dehydrogenases (ADH) enzymes (ADH1A, ADH1B, ADH1C, ADH4, ADH7), which are expressed in a tissue and time specific manner. The genes encoding these enzymes are clustered together in a 380 kb region on the long arm of chromosome 4. Genetic variation has been reported in these genes leading to differences in the ability to metabolise ethanol [9]. In slow metabolisers, peak alcohol levels may be higher and persist for longer than in fast metabolisers. It is hypothesized that alleles which result in “fast” metabolism of ethanol will protect against abnormal brain development in infants. The importance of peak blood alcohol concentration has been demonstrated in animal and human studies of neuro-behavioural outcomes in offspring exposed to ethanol during fetal life [10]–[12]. Until recently there had only been a handful of studies which have looked at associations of *ADH* genotypes and alcohol-related infant outcomes. These studies have tended to focus on mothers who drank heavily during pregnancy and possibly due to very small sample sizes have produced conflicting results [13]–[18].

The principal exposure to alcohol in young children is likely to have occurred during fetal life. If the alcohol dose reaching the fetus is influenced by *ADH* genotype as discussed above then it should be possible to detect genotype effects on cognitive outcomes. Such effects should only be seen in offspring of women who report alcohol use during pregnancy (assuming such reporting is accurate) and their presence if detected in moderate alcohol users would provide further evidence that even moderate alcohol use can adversely affect childhood cognition. Because we do not know the relative contributions of maternal and fetal enzymes in metabolising alcohol in fetal life and it is likely that both contribute to overall alcohol exposure, we studied the effects of both maternal and child genotype on childhood cognitive outcomes. Analyses were stratified according to whether mothers consumed low-to-moderate amounts of alcohol during pregnancy or whether they abstained. This was done to test our hypothesis that an association between *ADH* genotype and cognitive outcomes would only exist  =  in the former group, but would not be present in mothers who did not drink.

## Methods

### Ethics Statement

Ethical approval came from the Avon Longitudinal Study of Parents And Children (ALSPAC) Law and Ethics Committee (IRB 00003312) and the four Local Research Ethics Committees (LREC), Southmead, Frenchay and Bristol and Weston Health Authorities. Informed written consent was obtained from participants in this study, and from the parents of children in this study.

### Study Population

The Avon Longitudinal Study of Parents and Children (ALSPAC) is a population-based prospective study investigating environmental and other factors that affect the health and development of children. The study methods are described in detail on the study website (http://www.alspac.bris.ac.uk). In brief, pregnant women living in three health districts centred in and around the city of Bristol, England who had an expected date of delivery between the start of April 1991 and the end of December 1992 were eligible. 14,541, approximately 85% of those eligible [19], enrolled in the study, and of these, 13,822 (95%) had a singleton, live born child. Detailed information was obtained from the mother throughout pregnancy and information on both the mother and child has been collected at regular intervals, and is ongoing.

### Measurement of Alcohol Intake

At 18 weeks’ gestation women were asked to complete a questionnaire, which included questions on their average amount and frequency of alcohol consumption before the current pregnancy, during the first trimester and in the previous 2 weeks or at the time when they first felt the baby move. One drink was specified as one unit of alcohol (corresponding to an ethanol content of approximately 8 grams), and women were asked to recall their frequency of drinking as never, <1 unit/week, ≥1 unit/week, 1–2 units/day, 3–9 units/day, or 10+ units/day. Around 32 weeks of gestation women completed another questionnaire in which they were asked about their average weekday and weekend alcohol consumption, from which weekly intake was derived. Any woman who reported drinking even if it was less than 1 unit/week either in the first trimester or when they felt the baby first move was classified as drinking during pregnancy. Women who reported drinking some alcohol at 32 weeks were also classified as drinkers, however women for whom this questionnaire was missing were not excluded from the stratified analysis, but coded according to their drinking status during the first trimester and when the baby first moved. At approximately 18 and 32 weeks of pregnancy women were also asked on how days during the past month they had drank 2 pints of beer (or the equivalent amount of alcohol), any women who reported doing this on at least one occasion was classified as a binge drinker in our analysis of the association between genotype and binge drinking. We excluded 269 women who reported drinking >6 units per week at any point during pregnancy from our main stratified analyses, because we were interested in the effect of moderate alcohol intake of the mothers during pregnancy on child IQ scores rather than the effects of heavy drinking.

### Measurement of Cognition

Cognitive testing was carried-out during a clinic visit of children at age 8 year using a shortened version (which is described in detail elsewhere [20]) of the Wechsler Intelligence Scale for Children (WISC-III) from which an overall age adjusted total score was derived [21].

### Measurement of Potential Confounders

Data on selected characteristics from mothers and their partners was used to conduct a sensitivity analysis adjusting for potential confounding factors. Maternal age at delivery was calculated from dates of birth of the mother and baby. Other data were obtained by questionnaires administered to the mother during pregnancy. Family social class was derived as the highest social class of the mother or her partner, which was based on occupation and determined according to the 1991 British Office of Population Statistics classification. This was dichotomised as manual or lower versus higher. Mother’s education was dichotomised as at most Ordinary Level (O-level) or equivalent versus higher. The O-level was an exam-based qualification for students aged 14–16 years, which was replaced by the General Certificate of Secondary Education (GCSE) in 1988 in the UK. Further details are available at http://www.direct.gov.uk/en/EducationAndLearning/QualificationsExplained/DG_10039024.

### Genotyping

Ten Single Nucleotide Polymorphisms (SNPs) in 4 *ADH* genes (*ADH4* rs4699714, rs3763894, rs4148884, *ADH1A* rs2866151, rs975833, rs1229966, *AHD1B* rs2066701, rs4147536, rs1229984 and *ADH7* rs284779) were selected for genotyping on the basis of literature searches either because they had previously been shown to be associated with alcohol metabolism, intake or dependency or because they were haplotype tagging SNPs. SNPs were genotyped by KBioscience (http://www.kbioscience.co.uk) using the KASPar chemistry, a competitive allele-specific PCR system using FRET quencher cassette oligos (http://www.kbioscience.co.uk/genotyping/genotyping-chemistry.htm). Blind duplicates and Hardy-Weinberg equilibrium tests were used as quality control checks. Genotyping success rate was above 93.3% and error rate from duplicates was below 0.25% for all SNPs.

### Ethnicity of Participants

Women and children of white-European origin only were included to avoid population stratification, as many polymorphisms in the *ADH* genes differ markedly across different populations [22], and patterns of alcohol drinking are culturally dependent. Ethnicity was available from self-report or had been imputed from five genetic ancestry-informative markers [23].

### Statistical Analysis

Deviation of genotype counts from Hardy Weinberg equilibrium (HWE) was tested by Pearson’s chi^2^ test using the *genhwi* command in STATA. For all our rs1229984-outcome analyses we grouped rare homozygotes and heterozygotes together and assumed a dominant effect (as the minor allele frequency (MAF) for rs1229984 was <0.05), our previous analysis of this SNP on alcohol intake suggested this was appropriate [23]. All other SNPs were consistent with a per rare allele effect and so we report per allele effects. Associations between maternal genotype and drinking at 18 weeks of pregnancy have been presented in our earlier paper [23] we also looked at the effects of offspring genotype and offspring genotype adjusted by maternal genotype and maternal binge drinking at 18 weeks to determine whether any effects we detected were due to metabolism or due to changes in alcohol intake. We tested associations between overall WISC score at age 8 and genotype using linear regression models, as WISC score was found to be approximately normally distributed.

We looked at mother’s genotype and child’s genotype separately as we did not know which was more important in determining the exposure of the fetus to alcohol. This analysis was stratified by whether or not mothers reported drinking alcohol during pregnancy.

### Sensitivity Analyses

The potential for confounding was determined by carrying out sensitivity analyses in which our models were adjusted by the following factors; mother’s education, mothers’ smoking, gestational age of the child, age of the mother at delivery, mother’s marital status, parity, mother’s partners alcohol consumption, social class of the mother and her partner, and also by 5 ancestry informative markers with established population-specific allelic distributions–rs713598 and rs1726966 in *TAS2R38* [24], rs4988235 in *MCM6* [25], A44871G in *ASPM* [26] and rs930557 in *CPH1* [26].

### Stepwise Selection of Genetic Variants

Overall exposure to alcohol in utero is likely to be determined not by a single genetic variant in this region, but by several maternal and/or offspring genetic variants within this pathway. In order to account for this and for linkage disequilibrium in the area, which may lead to confounding between genotypes, we used Akaike’s Information Criterion [27] to carry out a backwards stepwise selection of all child genotypes based on WISC score at age 8, in which all genotypes were initially entered into the model and then eliminated in turn based onthe goodness of fit of the model, the purpose of which was to find the minimum set of markers which were associated with WISC score after taking into account linkage disequilibrium between the SNPs. Moreover, since offspring genotype is determined by alleles inherited from the mother and from the father and so will reflect mother’s genotype to a certain extent we repeated this stepwise selection including all mother and child genotypes in the model, to determine the minimal set of genetic variants (whether from the mother or the fetus) which were independently associated with overall WISC score at age 8.

### Linkage Disequilibrium Analysis

Pair-wise linkage disequilibrium (LD) across the SNPs was computed in mothers using haploview (http://www.broadinstitute.org/scientific-community/science/programs/medical-and-population-genetics/haploview/haploview) [28].

### Test of Interaction between Number of Risk Alleles and Alcohol Intake in the Mothers

We took the four SNPs which were selected as being related to WISC score among children using Akaike’s Information Criterion (*ADH7* rs284779, *ADH1B* rs4147536 *ADH1A* rs975833 and *ADH1A* rs2866151) and constructed a genotype score based on the number of rare alleles a child carried across the four loci, given that at each loci a child could carry zero, one or two rare alleles. We carried-out an analysis of this score on WISC at age 8 stratified by alcohol intake (any versus none) during pregnancy. When we had constructed our genotype score we found that most children (3939/4167, 94.5%) had a total of two, three or four ‘risk’ alleles across these sites, only eight out of 4167 children had no ‘risk’ alleles at this site, with 195 children having one risk allele and only 25 having five risk alleles, no one had more than five ‘risk” alleles. For our analysis, children were grouped as having less than or equal to two risk alleles, three risk alleles or greater than or equal to four alleles. We excluded mothers who reported drinking more than one unit per day during pregnancy, leaving 4167 women and their children who had provided sufficient data and were eligible for this analysis. The association between this score and total WISC score was tested using a linear regression model. In addition, an analysis of gene-environment interaction was carried out using this score and ever drank during pregnancy as the exposures and WISC score as the outcome. A likelihood ratio test was carried-out to compare a model with no interaction term against one with an interaction term.

## Results

14541 women were originally recruited into ALSPAC. However, we restricted our analysis to live singleton births, which were first ALSPAC births and excluded women and children of known non-white ethnic origin and those with missing ethnicity data, which left us with 11086 eligible mother-child pairs. Not all of these children and their mothers had data on all genotypes or on all observational variables. Numbers for the separate analyses are provided in the tables. The mean age-adjusted WISC score among the 6196 eligible children who completed the test at age 8 was 104.7 (SD = 16.3). This was slightly higher among those participants who also had genotype data available (mean = 105.1 SD = 16.2). Genotype distributions however, did not differ by whether participants also had alcohol data and WISC scores. The proportion of mothers drinking during pregnancy was higher in all those eligible for whom this data was available (77.6%, 7168/9236) compared to those who also had genotype data available (69%, 3066/4436).

HWE was assessed in all eligible individuals for whom genotype data was available. Three SNPs showed some evidence of being in Hardy Weinberg disequilibrium in either mothers or children, these were rs4699714 in mothers and rs3762894 and rs284779 in children. We examined the genotype calling by eye for these 3 SNPs to determine whether particular genotypes were more likely to be undetermined, or whether there appeared to be any miscalling but this was not the case. In addition we carried out a sensitivity analysis in which all samples with missing genotypes were coded as any 1 particular genotype, but as this could not explain the HWE we did not exclude any genotype on the basis of HWE. The A allele (48His) at rs1229984 was rare (<3%) in both children and mothers, however all other alleles were present at a frequency of at least 8%.

The ADH1B rs12229984 SNP among mothers was found to be associated with binge drinking at 18 weeks of pregnancy (as previously reported [23]). In addition, the rare alleles of ADH4 rs4699714 and ADH1B rs2066701 were more common among children of mothers who reported binge drinking at 18 weeks of pregnancy after adjustment for mothers genotype at these loci (OR = 1.17 (95% CI = 1.01–1.36), p = 0.03 and OR = 1.19 (95% CI = 1.03–1.38), p = 0.02 respectively).

Univariate analyses of mother and child genotypes and WISC score at age 8 (Table 1) stratified by whether mothers reported moderate alcohol use (<1–6 units per week) during pregnancy or not suggested a decrease in WISC score with the presence of the rare allele at *ADH7* SNP rs284779 among children and mothers. The rare allele at *ADH4* rs414884 in mothers was also associated with a decrease in IQ score among their children as was the rare allele at *ADH1A* rs2866151 and the common allele at rs1229966. All of these effects were only present among children whose mothers reported drinking during pregnancy. Among children of non-drinking mothers *ADH4* rs4699714 and rs4148884 were associated with WISC scores, and there seemed to be an effect of mother’s genotype at *ADH1B* rs1229984 although evidence for these effects was generally weak.

**Table 1:** Results for associations between mother and child genotypes and total WISC score at age 8.

Gene	SNP-rsnumber	Rare allele freq	HWEp-value	Mothers drinking (<1–6 units per week)During pregnancy			Mothers not drinking during pregnancy		
*Mother*				N	Per allele effect on WISC (SE)	P-value	N	Per allele effect on WISC (SE)	P-value
*ADH4*	rs4699714	0.27	0.05	2344	0.14 (0.52)	0.80	1203	−0.47 (0.73)	0.52
*ADH4*	rs3762894	0.16	0.44	2352	0.63 (0.64)	0.33	1195	0.08 (0.88)	0.93
*ADH4*	rs4148884	0.08	0.85	2351	−1.53 (0.84)	0.07	1206	−0.94 (1.16)	0.42
*ADH1A*	rs2866151	0.46	0.44	2304	−1.23 (0.48)	0.01	1181	0.54 (0.66)	0.41
*ADH1A*	rs975833	0.24	0.13	2323	0.93 (0.57)	0.10	1193	−0.49 (0.78)	0.53
*ADH1A*	rs1229966	0.36	0.22	2330	1.18 (0.50)	0.02	1191	−0.004 (0.69)	1.00
*ADH1B*	rs2066701	0.29	0.43	2294	0.43 (0.54)	0.42	1171	−0.003 (0.74)	1.00
*ADH1B*	rs4147536	0.22	0.70	2326	0.49 (0.58)	0.40	1193	0.179 (0.81)	0.83
*ADH1B*	rs1229984*group 2&3	0.03	0.57	2346	−1.27 (1.62)	0.43	1195	2.44 (1.94)	0.21
*ADH7*	rs284779	0.45	0.91	2343	−1.40 (0.48)	0.003	1200	0.77 (0.68)	0.26
***Child***									
*ADH4*	rs4699714	0.28	0.56	2962	−0.30 (0.47)	0.52	1456	−1.41 (0.66)	0.03
*ADH4*	rs3762894	0.17	0.01	2940	0.38 (0.55)	0.49	1458	0.09 (0.80)	0.91
*ADH4*	rs4148884	0.08	0.75	2959	0.21 (0.76)	0.78	1474	2.48 (1.06)	0.02
*ADH1A*	rs2866151	0.47	0.16	2903	−0.45 (0.42)	0.29	1433	0.08 (0.61)	0.89
*ADH1A*	rs975833	0.24	0.74	2926	−0.32 (0.50)	0.52	1450	−0.06 (0.69)	0.94
*ADH1A*	rs1229966	0.37	0.39	2933	0.40 (0.44)	0.36	1461	0.10 (0.62)	0.87
*ADH1B*	rs2066701	0.29	0.43	2886	0.02 (0.47)	0.97	1429	−0.06 (0.66)	0.92
*ADH1B*	rs4147536	0.20	0.49	2945	0.13 (0.53)	0.80	1453	−0.20 (0.73)	0.78
*ADH1B*	rs1229984*group 2&3	0.03	0.57	3364	−1.06 (1.26)	0.40	1702	2.02 (1.61)	0.21
*ADH7*	rs284779	0.45	0.01	2945	−1.27 (0.41)	0.002	1465	0.29 (0.60)	0.63

A linear regression model was used for this analysis, *Dominant effect.

Because overall in-utero exposure to alcohol will be determined by genotypes at several loci within ADH genes and by interactions between mother and child genotypes, and to account for confounding by linkage disequilibrium a backwards stepwise selection based on Akaike’s information criterion was used to select the best fitting genetic model for predicting child’s IQ. When only child’s genotypes were entered in the model, the following SNPs were identified as being independently associated with WISC score: *ADH1A* rs2866151, rs975833, *ADH7* rs284779 and *ADH1B* rs4147536. Further analyses to uncover the nature of interactions between genotypes at these SNPs found that the 2 SNPs in ADH1A were in complete linkage disequilibrium (LD)(D’ = 1, r^2^ = 0.28 chi^2^ = 1967 df = 1) with each other, such that individuals who had the rare homozygote genotype at one site always had the common homozygote genotype at the other site. However, individuals with no rare alleles at these 2 sites had a greater WISC score than those with any 1 rare allele with those having any 2 rare alleles (whether rare homozygotes at one site or heterozygotes at both sites) having the lowest WISC score. In addition, strong, but not complete, LD exists between these *ADH1A* sites and *ADH1B* rs4147536 (rs2866151- rs4147536, D' = −0.91, r^2^ = 0.18, > chi^2^ = 1284 df = 1, rs975833-rs4147536, D' = 0.95, r^2^ = 0.07, chi^2^ = 514, df = 1). Adding rs4147536 to a model containing both *ADH1A* SNPs strengthened the association with the *ADH1* SNPs, and we found that having any 3 alleles across the *ADH1A* and *ADH1B* SNPs rs2866151, rs975833 rs4147536 was associated with the lowest WISC score, although no-one in our study was found to have more than 3 rare alleles across these 3 sites. Rs284779 in *ADH7* is not in LD with the other 3 SNPs mentioned above, but it did show evidence of interaction with a composite score of the other 3 SNPs.

The results of 2 logistic regression models (one model including children of mother’s who drank during pregnancy and 1 including children of mothers who did not drink) with mutual adjustment for genotype at all of the four loci above are given in Table 2. When all mother and child genotypes were entered into the model the above 4 genotypes in the children were identified as important in predicting WISC score plus additionally *ADH4* rs4148884 in both the mothers and the children, results are not shown here, but can be provided on request. However, in this model the results of for *ADH4* rs4148884 were complex with the rare allele in mother’s being associated with a decrease in child’s IQ and the rare allele in children being associated with an increase in child’s IQ, as indicated in the unadjusted analyses in Table 1.

**Table 2:** Results for adjusted model including 4 child variants.

Gene	SNP	Maternal drinking during pregnancy			
		<1–6 units per week N = 2792		Non-drinkers N = 1375	
*Child*		Per allele effect on WISC score &95% confidence intervals	P-value	Per allele effect on WISC score &95% confidence intervals	P-value
*ADH1A*	rs2866151	−1.95(−3.29 to−0.61)	0.004	−0.38(−2.47 to 1.71)	0.72
*ADH1A*	rs975833	−1.72(−3.23 to −0.21)	0.03	−0.66(−2.90 to 1.59)	0.53
*ADH1B*	rs4147536	−1.47(−2.97 to 0.02)	0.05	−0.71(−2.92 to 1.50)	0.53
*ADH7*	rs284779	−1.27(−2.10 to −0.44)	0.003	−0.11(−1.12 to 1.35)	0.18

In our analysis of genotype score (based on adding together the number of rare alleles present across 4 SNPs listed in Table 4) and WISC (Table 3), we found strong evidence of a dose-response between number of risk alleles and total WISC score (Effect estimate −1.20 (95% CI −1.89 to −0.52) per allele p = 0.001), with WISC score decreasing with increasing number of risk alleles. We found that this effect was limited to the children of mothers who reported drinking during pregnancy (Effect estimate  = −1.80 (95% CI =  −2.63 to −0.97) p = 2×10^−5^), and there was evidence of an interaction between number of risk alleles and mother’s drinking behaviour on WISC score at age 8 (p_interaction_ = 0.009). In addition, the effect of genotype score among children of drinking mothers was strengthened after adjustment for amount drank at 32 weeks of pregnancy (adjusted effect estimate  = −2.65(95% CI = −3.75 to −1.54) p = 2.8×10^−6^).

**Table 3:** IQ score by number of risk alleles stratified by maternal alcohol intake during pregnancy.

	Number of risk alleles*			Effect estimateand 95% CI	P-value (dose response)
Mother’s drinking status	≤2	3	≥4		
Non-drinkers	N = 519 Mean = 103.1 (SD = 16.7)	N = 628 103.5 (15.7)	N = 228 103.2 (15.8)	0.16 (−1.05 to 1.36)	0.80
Drinking during pregnancy(<1–6 units per week)	N = 1139 107.5 (16.3)	N = 1171 105.4 (16.1)	N = 482 104.0 (15.8)	−1.80 (−2.63 to −0.97)	0.00002
All women	N = 1658 106.1 (16.6)	N = 1799 104.7 (16.0)	N = 710 103.8 (15.8)	−1.20 (−1.89 to −0.52)	0.001

P- value for interaction between number of risk alleles and drinking during pregnancy  = 0.009

* Total number of risk alleles in *ADH1A* rs2866151 rs975833, *ADH1B* rs4147536 and ADH7 rs284779.

**Table 4:** Mothers age, educational level and socio-economic group by genotype score and drinking behaviour during pregnancy.

	Genotype scoreNumber of risk alleles*			Mothers drinking during pregnancy	
Mother’s charateristics	≤2	3	≥4	<1–6 units per week	Abstainers
Age (years)	29.2±4.6	29.2±4.3	29.8±4.5	29.7±4.4	28.4±4.5
Education(%O-level or higher )	44.6	43.4	43.4	47.0	37.5
Socio-economic group(% Non-manual occupation)	58.7	57.7	55.7	61.7	51.8

This effect did not change in a sensitivity analysis excluding all women who reported binge drinking (≥4 units of alcohol) either at 18 weeks or 32 weeks of pregnancy (question asked at these time points but refers to the preceding month) (p = 0.005). For all categories of allele score, drinking during pregnancy was associated with a higher IQ score in the child. We also looked at the effect of genotype score on offspring IQ among the 269 women excluded from our main analysis due to drinking >6 units per week during pregnancy. 192 of these women drank 1–2 units per day during pregnancy and 77 women drank more than 2 units per day during pregnancy. We found that among those women drinking 1–2 units per day there was no evidence that the effect of genotype score was any different from mothers drinking <1 unit per day (per allele effects for these women were −1.08, 95% CI = −4.23 to 2.06 p = 0.5), whereas for the 77 women drinking more than 2 units per day the effect of genotype appeared to be double that among moderate drinkers (per allele effect −3.52 95% CI = −7.96 to 0.93, p = 0.12). However, reflecting the small number of women in these groups, the evidence for these effects is weak and the confidence intervals are wide.

We did not find evidence of an association between fetal genotype score and alcohol intake among the pregnant women in this study, suggesting that the above effects are due to metabolism of alcohol rather than alcohol intake.

Adjustment of analyses for five ancestry informative markers, and adjustment for potential confounders made no difference to the results (data not shown), but these variables have previously been shown not to be associated with the genotypes analysed here [23].

Table 4 shows mothers age, educational level and socio-economic group according to drinking status and child’s genotype score, for the mothers with data on both. Mothers who drank moderately during pregnancy were older, better educated and less likely to be from a manual socio-economic group. However, these factors were not associated with genotype score.

## Discussion

### Purpose of the Study and Overall Result

Observational studies have suggested that whilst heavy alcohol drinking during pregnancy causes fetal alcohol syndrome, there are no apparent adverse effects associated with moderate drinking [3], [4]. This has led to a disparity between scientific evidence and current advice given to pregnant women. The purpose of this study was to determine whether exposure to moderate levels of alcohol during gestation influences child’s cognition. We investigated associations between genetic variants in alcohol metabolising enzymes in mothers and their children, in a population based study of women, many of whom drank in moderation during pregnancy to determine whether these variants were related to IQ scores in the child at age 8. We found strong evidence that four such variants among children were related to differences in IQ and that this effect was only present in children of mothers who drank some alcohol during pregnancy. Such gene-environment interactions can be taken as providing evidence supporting a causal association with the outcome [29], although causation cannot be proven by the current study design. Additionally another genetic variant in *ADH4* in mothers was associated with their child’s IQ.

### Genetic Variation in ADH Genes and Alcohol Metabolism

There is considerable between-individual variation in blood alcohol concentrations achieved following ingestion of a standard weight-adjusted amount of alcohol [30]. Variation in the *ADH* region is thought to contribute substantially to this variation [9], with the largest effect on breath and blood alcohol levels due to enzymes which act early in the time course of alcohol metabolism, soon after ingestion when ethanol concentrations are highest [9]. Another recent study by Birley [31] has identified a region in *ADH7* as being the one most strongly associated with alcohol metabolism, when examining the whole of the *ADH* region. Genetic variation in *ADH1A, ADH1B* and possibly *ADH4* were also shown in the same study to affect the early or first-pass phase of alcohol metabolism. *ADH7* is expressed in the stomach mucosa [32] and acts early to metabolise alcohol before it reaches the blood. Interestingly, in rats during pregnancy overall ethanol elimination rates were increased and it was gastric ADH enzymes, as opposed to hepatic enzymes which showed elevated expression [33]. *ADH1A* is particularly interesting in relation to fetal exposure to alcohol as this is expressed from the first trimester of fetal life becoming less active later in gestation and only weakly active during adult life [34]. *ADH1B* is also active in the liver from the second trimester and gradually increases in activity, such that in adults this locus is responsible for most of the liver ADH activity [35]. *ADH4* is expressed in the liver and may account for 40% of alcohol oxidation at intoxicating levels [36]. There are very few extensive studies of SNPs in *ADH* genes and alcohol metabolism, thus the relevant polymorphisms (apart from a couple of rare exceptions) along with the direction and size of effect is still not clear. However, we have summarized what is known about the genetic variants we selected in Text Box S1.

### Genetic Variation and Offspring IQ Score

Given the level of complexity of this pathway and the amount of redundancy due to the fact that 5 *ADH* genes are all catalysing the same reaction, we hypothesised that any one SNP would have only a minor effect on metabolism of alcohol and that interactions between SNPs across this region would be important. Previous studies have shown that interactions between SNPs across these genes are apparent in the risk of alcoholism, in particular between *ADH1B* and *ADH7* and between *ADH4* and *ADH1A* [40], [41]. In line with this a multivariate model which included several SNPs across this region showed that after adjustment for variants at other sites rare alleles at *ADH1A* rs2866151 and rs975833, *ADH1B* rs4147536 and *ADH7* rs284779 among children were associated with decreased IQ scores at age 8, whereas, aside from the SNP in *ADH7*, these SNPs were not associated with IQ in univariate models suggesting interactions between SNPs. When mother’s genotype as well as child’s genotype was entered into the model *ADH4* rs4148884 was also important with opposing effects for mother and child genotype at this loci.

The mutually adjusted effects at *ADH1A* rs2866151 and rs975833 and ADH1B rs4147536 are greater than the unadjusted effects at this site. The explanation for this could be that there is evidence of strong linkage disequilibrium (possibly due to selection) across these loci which means that individuals in this study did not have more than three out of six possible rare allele across the three sites, this coupled with the finding that there seems to be an additive effect across the three sites such that individuals with no rare alleles had the highest WISC score followed by those with any 1 rare allele, followed by those with any two rare alleles and those with any three rare alleles had the lowest score. *ADH7* rs284779 was not in LD with the above SNPs but there was evidence of an interaction between the *ADH7* SNP and SNPs at *ADH1A* and *ADH1B.*

### Relative Importance of Fetal and Maternal Genotypes on Fetal Exposure to Alcohol

There is very little published on the relative importance of fetal and maternal ADH enzymes in determining alcohol exposure of the fetus. We anticipated that enzymes in both the fetus and the mother may play a role, thus we looked at the effect of maternal genetic variation, fetal genetic variation, and because the fetal genotype is a combination of alleles inherited from both the mother and the father, we used stepwise selection of all maternal and fetal genotypes to determine which (mother or child) were having an independent effect on child’s IQ. Most of the genetic variants which we found to predict child’s IQ among offspring exposed to alcohol were child genotypes. This is in line with evidence that *ADH1A* is expressed at high levels in the fetus with *ADH1B* being expressed from the second trimester onwards with very minor roles for these enzymes in alcohol metabolism later in life [34].

### Consistency with Previous Studies

To our knowledge this is the first population-based study to investigate the role of *ADH* variants in children and their mothers in a study in which most of the mother’s drank in moderation during pregnancy. A handful of very small studies have been carried-out among heavy drinking mothers and their children to look at the effects of ADH enzymes on the presence of fetal alcohol syndrome with mixed results due to a lack of power [13]–[19]. Many studies including our own have looked at the association between maternal alcohol intake measured by questionnaire and offspring IQ [42]–[44] most showing as we do in Alati et al [43] that moderate alcohol intake during pregnancy is associated with an increase in child’s IQ relative to not drinking. However, moderate drinking in our study was found to be strongly associated with an increase in maternal age, increase in maternal education level and a higher social class all of which are associated with a higher IQ among children. Thus observed associations are probably due to confounding by socio-economically clustered factors. Conversely genotype is not on the whole associated with these factors and therefore associations between genotype and IQ are unlikely to be confounded by lifestyle factors [8].

Once we had excluded women with missing data (the main reason being lack of DNA) we were left with a much smaller subset of the original ALSPAC study. However our genotype and our WISC scores correspond well with larger samples of women with any of the above data. In addition, whilst missing exposure or phenotype data could possibly bias observational results, for example if there were a tendency for women who drank alcohol not to take part in the study or to be lost to follow-up (which is what we found), then this may bias any association between alcohol intake and child’s IQ. However, such selection is unlikely to have biased the genotype results as genotype is not associated with missingness.

One concern when using genetic variants to make inferences about associations between exposures and outcomes is pleiotropy [45]. This is the phenomenon whereby the gene may act on a number of pathways and thus may influence an outcome by a mechanism other than that involving the exposure of interest. ADH enzymes are involved in the metabolism of retinoic acid, a compound which is extremely important in fetal development. In addition, as well as influencing alcohol levels, variants in *ADH* will also have an effect on acetaldehyde levels, the primary substrate of alcohol. However, whilst the precise mechanism by which *ADH* genes influence child’s cognition needs further investigation the finding that these effects are limited to children of women who drank during pregnancy indicates that alcohol is the important exposure in this pathway. It is not established whether the genetic variants of *ADH* which were associated with child’s IQ in this study are associated with “fast” or “slow” metabolism of alcohol. We assume, based on the results of this study and on our previous work that these variants predispose to “slow” alcohol metabolism.

### Public Health Implications

If real these results could have important public health consequences, because cognitive ability has implications for social trajectories and health. It is well documented that individuals with lower IQ have lower socio-economic positions and poorer adult health and even higher mortality rates compared with those with higher IQs [46]–[48]. Whilst the effects of genotype appear modest, 3.5 points difference on the WISC scale for those children with ≤2 risk alleles compared to those with 4+ risk alleles, it is important to remember that these are effects for genotypes which are likely to result in very small differences in peak alcohol levels and alcohol exposure, and these subtle metabolic effects are among women drinking less than 1 unit of alcohol per day. Larger causal effects are anticipated for more substantial differences in fetal alcohol exposure levels, for example the differences existing between offspring of mothers with moderate alcohol consumption and mothers abstaining.

### Conclusion

Five variants in genes involved in alcohol metabolism amongst children and their mothers were associated with child’s cognitive ability at age 8 in a population-based study.

Associations between child’s genotype and outcome were only present among those whose mothers reported drinking alcohol in moderation during pregnancy. This suggests that, even amongst women drinking moderate amounts of alcohol, subtle changes in exposure to alcohol due to an ability to metabolise the substrate may be important, and offers some support to the hypothesis that even small amounts of alcohol in utero have an effect on future cognitive outcomes.

## Supporting Information

Text Box S1 (DOCX)
