# Supplementary material for: Fetal Alcohol Exposure and IQ at Age 8: Evidence from a Population-Based Birth-Cohort Study
Source: PLoS One. 2012 Nov 14;7(11):e49407. doi: 10.1371/journal.pone.0049407 (PMC3498109; doi:10.1371/journal.pone.0049407)
Supplement: Text Box S1 — (DOCX) [file pone.0049407.s001.docx]

**What is currently known about these genetic variants**

The rare allele at rs1229984 which encodes for an enzyme that metabolises alcohol at a much faster rate than the common allele at this locus has recently been associated with reduced alcohol intake among Australian twins [37]and among the pregnant women included in this study [23].

SNPs in ADH4 have been shown to be associated with alcohol and drug dependence in an extensive study of 110 SNPs across the ADH region. The ADH4 SNPs genotyped for this study were the 3 hapolotype tagging SNPs which captured variation across ADH4 responsible for this effect [38].

ADH1A rs2866151 and ADH7 rs284779 have been found to be associated with alcohol dependence [38], as have ADH1A rs975833 and rs1229966 [39].

Rs2066701 and rs4147536 in ADH1B were chosen for inclusion in the study because these were identified as being sufficient to capture much of the variation in the ADH1B gene (Hapmap).
